# Volitional muscle activity paired with transcranial magnetic stimulation increases corticospinal excitability

**DOI:** 10.3389/fnins.2014.00442

**Published:** 2015-01-12

**Authors:** Matthew A. Edwardson, David H. Avery, Eberhard E. Fetz

**Affiliations:** ^1^Department of Neurology, University of WashingtonSeattle, WA, USA; ^2^Department of Psychiatry and Behavioral Sciences, University of WashingtonSeattle, WA, USA; ^3^Department of Physiology and Biophysics, University of WashingtonSeattle, WA, USA

**Keywords:** transcranial magnetic stimulation, activity-dependent stimulation, timing-dependent plasticity, motor cortex, EMG-triggered, abductor pollicis brevis, extensor digitorum

## Abstract

Studies of activity-dependent stimulation in non-human primates suggest that pairing each instance of volitional muscle activity with immediate intracortical stimulation causes long-term-potentiation-like effects. This technique holds promise for clinical rehabilitation, yet few investigators have tested activity-dependent stimulation in human subjects. In addition, no one has studied activity-dependent stimulation on the cortical representation for two separate target muscles in human subjects. We hypothesized that 40 min of transcranial magnetic stimulation (TMS) triggered from ballistic muscle activity at a mean repetition rate of 1 Hz would cause greater increases in corticospinal excitability than TMS-cued muscle activity, and that these changes would be specific to the muscle of study. Ten healthy human subjects participated in 4 separate sessions in this crossover study: (1) visually cued volitional activation of the abductor pollicis brevis (APB) muscle triggering TMS (APB-Triggered TMS), (2) volitional activation of APB in response to TMS delivered from a recording of the prior APB-Triggered TMS session (TMS-Cued APB), (3) visually cued volitional activation of the extensor digitorum (ED) triggering TMS (ED-Triggered TMS), and (4) volitional activation of ED in response to TMS delivered from a recording of the prior ED-Triggered TMS session (TMS-Cued ED). Contrary to our hypothesis, we discovered evidence of increased corticospinal excitability for all conditions as measured by change in area of the motor evoked potential. We conclude that single TMS pulses paired either before or after muscle activity may increase corticospinal excitability and that further studies are needed to clarify the optimal time window for inducing neural plasticity with activity-dependent stimulation. These findings will inform the design of future activity-dependent stimulation protocols for clinical rehabilitation.

## Introduction

Recent advances in our understanding of synaptic plasticity indicate that appropriately timed delivery of brain stimulation can alter the strength of neural connections (Caporale and Dan, 2008; Kleim, 2010). *In vitro* experiments show that connections between two neurons are strengthened when the postsynaptic neuron fires at a short interval after presynaptic input (Markram et al., 1997; Bi and Poo, 1998). The concept that “neurons that fire together wire together” was first postulated by Hebb (1949), and provides the framework for manipulating synaptic plasticity with activity-dependent stimulation. Both animal (Jackson et al., 2006; Rebesco et al., 2010; Lucas and Fetz, 2013; Nishimura et al., 2013) and human (Stefan et al., 2000; Bütefisch et al., 2004; Thabit et al., 2010) studies demonstrate that administering brain stimulation according to principles of spike-timing dependent plasticity (STDP) alters the organization of neurons in the primary motor cortex (M1). Targeting this neural reorganization to specific motor representations of interest holds promise for reducing disability in patients with brain injury (Edwardson et al., 2013).

Translating activity-dependent stimulation protocols from animals to human subjects required a transition from invasive to non-invasive stimulation techniques. The prior animal studies demonstrating evidence of motor plasticity used intracortical microstimulation (ICMS). ICMS allows for activation of small populations of neurons, but the technique is too invasive to employ in human subjects. Transcranial magnetic stimulation (TMS) on the other hand provides a non-invasive if less specific means by which to activate neural populations. Stefan and colleagues used TMS in an activity-dependent manner in humans by pairing TMS and peripheral nerve stimulation in a technique called paired associative stimulation (PAS) (Stefan et al., 2000). They observed evidence of increased corticospinal excitability in the form of larger amplitude motor evoked potentials (MEPs) and prolonged cortical silent periods (CSPs), but only when the pairs of stimuli were timed to arrive in M1 synchronously. This increased corticospinal excitability was thought to reflect a neuronal process similar to long-term potentiation (LTP) given the required specificity of the inputs and long-lasting effects.

Subsequent studies of activity-dependent stimulation paired volitional muscle activity with TMS, and may be better suited for translation given the similarities to current clinical rehabilitation strategies. Several questions remain prior to using such strategies on patients with brain injury. These studies conflict on whether LTP-like effects are best induced when muscle activity occurs before or after brain stimulation. Bütefisch and colleagues repeatedly paired muscle activity with subsequent brain stimulation and changed the direction of a TMS-evoked thumb movement (Bütefisch et al., 2004). On the other hand, a recent study by Thabit and colleagues used a reaction time task to vary the timing at which TMS was paired in relation to muscle activity (at −100 ms, −50 ms, +50 ms, +100 ms, and +150 ms intervals) and showed increases in MEP amplitude and prolonged CSP only when muscle activity occurred 50 ms *after* brain stimulation (Thabit et al., 2010). In contrast to the study by Bütefisch and colleagues, muscle activation 50 ms before TMS had no effect. Of note, both of these studies focused on the cortical representation for the abductor pollicis brevis (APB) muscle; whether the results generalize to other muscle representations such as the extensor digitorum (ED) that are particularly pertinent for clinical rehabilitation remains unclear. Stroke patients, for example, often have difficulty activating extensor muscles in the hand and wrist, limiting their ability to perform activities of daily living. The optimal stimulation intensity—high enough to induce plasticity, yet low enough to maintain specificity for the motor representation of interest—remains in question. Bütefisch and colleagues employed a low stimulation intensity of 80% resting motor threshold (RMT), whereas Thabit and colleagues used a higher intensity of 120% RMT which is more in line with PAS studies (Stefan et al., 2000; Wolters et al., 2003). More studies of activity-dependent stimulation in human subjects are needed to clarify the optimal timing, stimulation intensity, and whether LTP-like effects can be targeted to particular muscle representations.

The present study tests the hypothesis that repeatedly pairing volitional muscle activity with an immediately subsequent TMS pulse would produce greater increases in corticospinal excitability than a TMS pulse shortly before muscle activity in healthy human subjects. The primary outcome measures to assess corticospinal excitability include MEP area and CSP. We sought to confirm the evidence in non-human primate (Jackson et al., 2006; Lucas and Fetz, 2013) and human (Bütefisch et al., 2004) studies suggesting that repeatedly pairing muscle or cortical activity 0–50 ms before brain stimulation leads to LTP-like effects. In addition, the present study was designed to extend the work of Bütefisch et al. (2004) by demonstrating that LTP-like effects could be targeted to the cortical representation for specific muscles in human subjects, making activity-dependent stimulation more useful for clinical rehabilitation. While our hypothesis goes against the timing effects described by Thabit et al. (2010), theirs is the only study suggesting LTP-like effects when TMS precedes muscle activity. Our TMS-cued EMG sessions were designed as control sessions, as typical reaction times in humans range from approximately 90–150 ms (Pascual-Leone et al., 1992), which is well beyond the temporal window for potentiating effects described by Thabit et al. Thabit and colleagues also used a fairly high stimulation intensity of 120% RMT during conditioning (Thabit et al., 2010); we wanted to confirm evidence from Bütefisch and colleagues that a subthreshold stimulation intensity was capable of inducing LTP-like effects (Bütefisch et al., 2004) as this would reduce the very small but not negligible risk of seizure activity. We used a higher average stimulation frequency (1 Hz) during conditioning than Bütefisch et al. (0.1 Hz) or Thabit et al. (0.2 Hz), thinking this would increase the opportunity for Hebbian-type plasticity. In order to demonstrate an LTP-like phenomenon potentially useful for clinical rehabilitation, we sought evidence of increased corticospinal excitability that was: (1) specific to the muscle of study; (2) dependent on the timing of TMS in relation to volitional muscle activity; and (3) long-lasting. We tested our hypothesis on cortical sites for two different muscles, the APB and ED, at a mean pairing frequency of 1 Hz over 40 min at active motor threshold (AMT) intensity, expecting increases in corticospinal excitability specific to each muscle for the EMG-triggered TMS conditions.

## Materials and methods

### Subjects

Ten healthy volunteers (4 female, 6 male; mean age 24.0; age range 19–47) fulfilled inclusion/exclusion criteria (age >18, no history of seizures, no family history of seizures, no metal in the body, no history of elevated intracranial pressure, no major medical problems including neurologic or psychiatric disease, not pregnant, not taking neuroleptics or tricyclic antidepressants), gave written informed consent, and completed the study protocol that was approved by the Institutional Review Board of the University of Washington. An Investigational Device Exemption was granted by the US Food and Drug Administration for activity-dependent stimulation. All subjects were right-handed according to the Oldfield handedness inventory (Oldfield, 1971).

### Equipment

TMS was performed with a Dantec MagPro with 70 mm figure-of-eight coil. Surface electromyographic (EMG) activity from relevant forearm muscles was amplified and bandpass filtered at 20–450 Hz (4-Channel Bagnoli, Delsys Inc., Boston MA), sampled at 2 kHz using a data acquisition device (National Instruments Inc, Austin TX), and stored on a laptop computer. A custom LabView program processed real-time EMG activity and delivered triggering pulses via transistor-transistor logic to the Dantec MagPro.

### Experimental procedures

**Figure 1:**
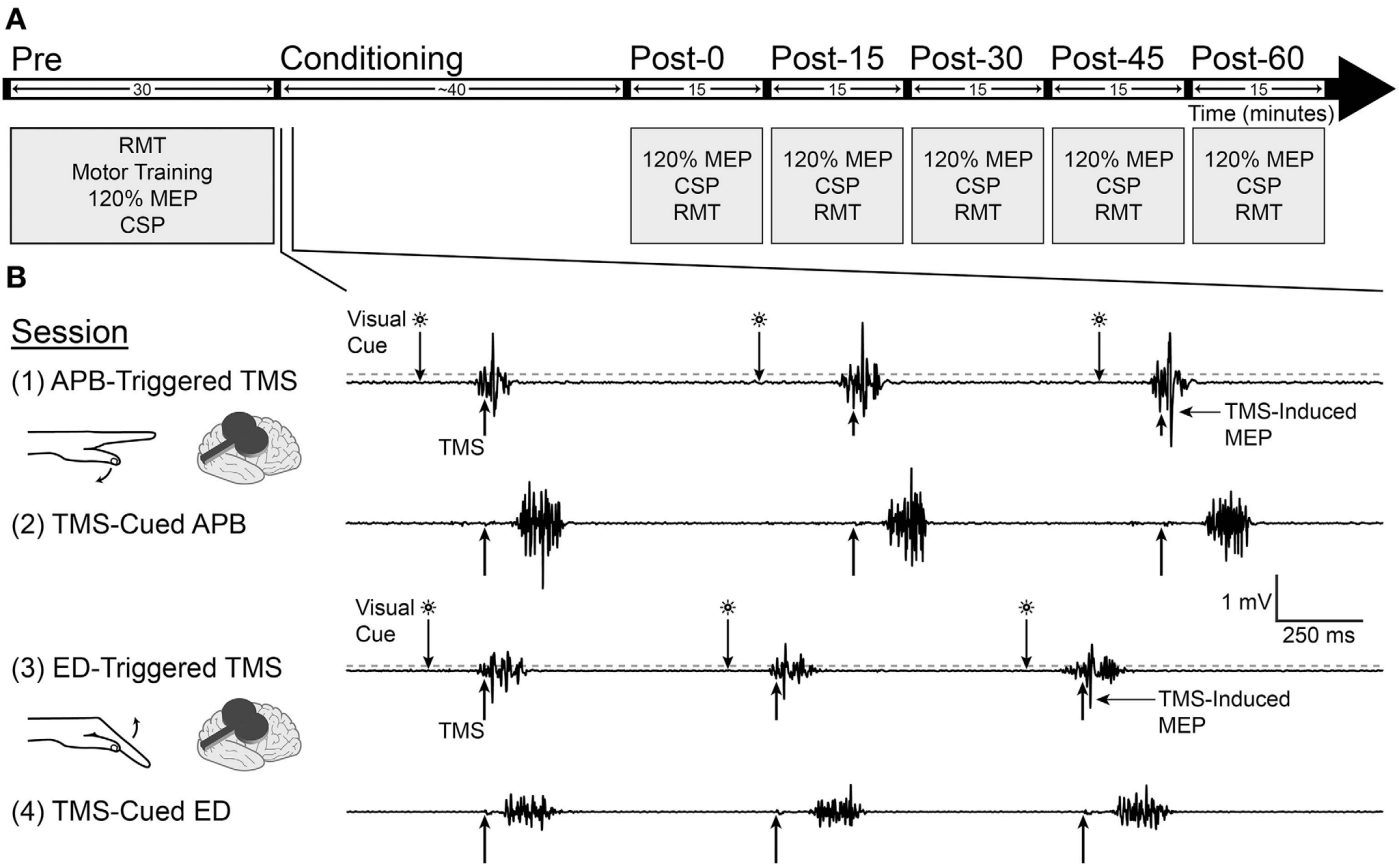
**Experimental design**. **(A)** Measurements of motor evoked potential (MEP) area under the curve at 120% resting motor threshold (RMT), cortical silent period (CSP), and RMT were made before and after conditioning for each study session. **(B)** Four separate conditioning sessions took place on successive weeks, involving the abductor pollicis brevis (APB) in sessions 1 and 2 and extensor digitorum (ED) in sessions 3 and 4. In the APB-triggered TMS and ED-triggered TMS sessions, muscle flexion in reaction to a visual cue triggered TMS when EMG amplitude rose above a predefined threshold (200 μV for APB and 100 μV for ED, denoted by dashed gray line). In the TMS-cued APB and TMS-cued ED sessions, a computer delivered TMS pulses with timing identical to the prior session; subjects activated the muscle as soon as possible after feeling or hearing the TMS pulse. Recordings are from one representative subject during conditioning.

Subjects served as their own controls in this repeated measures, crossover study. Each subject participated in 4 study sessions separated by at least 1 week. The experimental protocol is shown in Figure 1. The goal duration for all conditioning sessions was 40 min. This duration was based on data from a study in non-human primates suggesting that at least 20 min of EMG-triggered intracortical microstimulation was necessary to produce neural plasticity (Lucas and Fetz, 2013). The goal mean pairing frequency for all study sessions was 1 Hz. The rationale for this frequency was that preliminary data using a pairing frequency of 0.1 Hz (unpublished) showed no difference in corticospinal excitability between the EMG-triggered and TMS-cued conditions. We expected that increasing the pairing frequency to 1 Hz would maximize the number of pairings between TMS and volitional muscle activity, thereby increasing the opportunity for neuroplastic change. Surface EMG was recorded from APB, ED, flexor carpi radialis (FCR), and biceps in the non-dominant arm. FCR and biceps served as control muscles during experimental procedures. The site for the ED electrode was marked at the midway point between the lateral epicondyle of the humerus and Lister's tubercle at the wrist. The APB muscle was chosen because a prior activity-dependent TMS study demonstrated motor plasticity in the cortical representation for APB (Bütefisch et al., 2004). The ED muscle was chosen because patients with neurologic injury often have difficulty activating the finger extensors, so targeting the ED for neuroplastic change would be relevant to promoting functional recovery in future neurorehabilitation interventions.

### Resting and active motor threshold determination

At the beginning of each study session a lycra swim cap specific to each subject was placed over the scalp. The subject reclined in a study chair custom fit with a concave memory foam cushion placed behind the head. With the TMS coil placed tangential to the scalp and angled 45°C from midline, single TMS pulses were delivered over non-dominant M1. The motor hotspot for the muscle of study (APB or ED) was determined by finding the site where TMS produced the highest amplitude MEPs for each respective muscle, which was marked on the lycra cap. This hotspot was used as the target site for the remainder of the session. Manual hotspot determination was repeated at the beginning of subsequent study sessions, sometimes yielding slightly different hotspot locations. The RMT and AMT were recorded for the APB and ED muscles. RMT was defined as the intensity at which more than 5 of 10 MEPs were larger than 50 μV in amplitude with the target muscle at rest (Maeda et al., 2000). AMT was defined as the intensity at which more than 5 of 10 MEPs were larger than 200 μV (Rothwell et al., 1999) in amplitude while subjects held isometric contraction at 200 μV for APB or 100 μV for ED; this contraction corresponded to approximately 10–20% maximal EMG amplitude for each muscle respectively.

### Motor training

Subjects practiced ballistic contraction of the muscle of study in response to the appropriate cue for 5 min at the beginning of each study session. The cue was a flashing green light on the computer screen (for EMG-triggered sessions) or the sound of the TMS device discharging (with the coil held away from the scalp during motor training) at time intervals identical to the first 5 min of the previously recorded EMG-triggered conditioning session (for TMS-cued sessions). Motor training continued until the subject could consistently respond < 400 ms after the cue, generate a uniform amount of muscle activity (goal amplitude of 1000 μV for APB or 500 μV for ED), and suppress muscle activity between contractions. Subjects received real-time visual feedback on a computer screen in the form of EMG tracings from all muscles as well as a vertical line appearing at TMS onset. For all practice and study sessions subjects were instructed to perform a single, ballistic contraction and relaxation of the muscle of study in response to each appropriate cue while holding the forearm in pronation. The mean maximum EMG amplitude ± SD during motor training was 1.00 ± 0.39 mV for APB-triggered TMS, 0.98 ± 0.34 mV for TMS-cued APB, 0.45 ± 0.14 mV for ED-triggered TMS, and 0.47 ± 0.13 mV for TMS-cued ED.

### MEP area under the curve, cortical silent period, and RMT measurements

MEP area under the curve (AUC), cortical silent period (CSP), and RMT were measured before, immediately after, and at 15 min intervals post-conditioning for 1 h to document changes in corticospinal excitability. For these measurements single-pulse TMS was delivered 10 times at 10 s ± 1 s intervals; the frequency varied in a box-car distribution over the 9–11 s interval to discourage anticipation. MEP AUC was defined as the average AUC of 10 rectified MEPs at 120% pre-conditioning RMT (Maeda et al., 2000) (**Figure 3**). We planned to use the EMG recordings from the control muscles (FCR, biceps) to determine whether changes in MEP AUC were specific to the muscle of study. CSP was defined as the average duration of 10 silent periods at 130% pre-conditioning RMT, as measured from MEP onset to the return of sustained EMG while subjects held isometric contraction at the same effort used for pre-conditioning AMT measurements (Damron et al., 2008) (**Figure 6**).

### Conditioning sessions (Figure 1B)

#### APB-triggered TMS (session 1)

Subjects performed a single ballistic contraction of the APB muscle each time they saw a green light flash on the computer screen. In session 1, APB EMG amplitude above 200 μV triggered a single TMS pulse at pre-conditioning APB AMT intensity (mean 86.2% RMT) to the contralateral APB hotspot.

#### TMS-cued APB (session 2)

Using a recording of the TMS delivery times for the subject's previous APB-Triggered TMS session, a computer triggered single TMS pulses at pre-conditioning APB AMT intensity (mean 83.9% RMT) with timing identical to the previous APB-Triggered TMS session. Subjects were instructed to use all sensory modalities available (sight of TMS pulse appearing as a line on the computer screen, sound of stimulator, tactile sensation from scalp) to perform a single ballistic contraction of the APB muscle as quickly as possible following each TMS pulse and try not to anticipate any stimuli, to reduce premature responses.

#### ED-triggered TMS (session 3)

ED-triggered TMS was identical to APB-triggered TMS, except that subjects were instructed to perform ballistic extension of the fingers in response to each visual cue, EMG was recorded from the ED muscle, and TMS was delivered to the contralateral ED hotspot at pre-conditioning ED AMT intensity (mean 87% RMT) when ED EMG amplitude rose above 100 μV.

#### TMS-cued ED (session 4)

TMS-cued ED was identical to TMS-cued APB, except that TMS was delivered to the contralateral ED hotspot at pre-conditioning ED AMT intensity (mean 88.3% RMT) at TMS delivery times identical to the previous ED-Triggered TMS session. Subjects were instructed to perform a single ballistic extension of the fingers following each TMS pulse and try not to anticipate any stimuli.

### Stimulation parameters

During conditioning with muscle-triggered TMS, subjects received 1 visual cue per second ± 0.1 s as a signal to activate the muscle. However, if the subject responded late to a visual cue the subsequent cues were delivered slightly faster, according to a computer algorithm in order to achieve a mean stimulation frequency of 1 Hz. During conditioning with TMS-cued muscle activation, subjects received TMS using a recording of the exact TMS delivery times from the prior session. Note that inter-stimulus intervals (ISI) during TMS-cued muscle activity were not uniform—they were irregular, just as they were during the prior EMG-triggered session reflecting the reaction times to the visual cue during EMG-triggered TMS. The mean ISI was 1.00 ± 0.17 s for the APB-triggered TMS and TMS-cued APB sessions and 1.00 ± 0.12 s for the ED-triggered TMS and TMS-cued ED sessions. 30 s inter-train intervals were introduced between stimulus trains during EMG-triggered TMS to reduce seizure risk and muscle fatigue. These inter-train intervals were also experienced by subjects during TMS-cued EMG sessions since stimulation was based on a recording of delivery times from EMG-triggered TMS. The duration of each stimulus train depended on the duration of each ISI and was derived from an algorithm based on the safety data for TMS subjects at rest (Chen et al., 1997) as follows. Each ISI was converted into 1/the maximum number of allowable pulses for the corresponding frequency (see Table 1) and added to a variable *X* according to the equation *X* = *X* + (*1/pulses*). When *X* reached 1, a 30 s inter-train interval was introduced, and *X* was reset to 0 prior to the next stimulus train. ISIs representing frequencies not provided in the safety table were rounded to the next highest frequency to derive 1/pulses. The mean duration of stimulus trains ± SD was 77 s ± 7 s for APB-triggered TMS and TMS-cued APB sessions, and 78 s ± 6 s for ED-triggered TMS and TMS-cued ED sessions. The mean conditioning duration ± SD was 39.7 ± 1.1 min for APB-triggered TMS and TMS-cued APB and 39.7 ± 0.8 min for ED-triggered TMS and TMS-cued ED.

**Table 1:** **Safe maximum duration and number of pulses for individual TMS trains at resting motor threshold (Chen et al., 1997)**.

**Frequency (Hz)**	**Duration (s)**	**Pulses**	**1/Pulses**
1	>270	>270	0.0037
5	10	50	0.02

### Statistics

Statistical analysis was performed with ANOVA and two-tailed Student's *t*-tests. All ANOVA statistics were Two-Way, repeated-measures, reported as *F_x, y_* = value, where *x* and *y* represent degrees of freedom within groups.

To determine the variability of the pre-conditioning AMT, RMT, MEP AUC, and CSP measures from session to session, we performed dependent means *t*-tests between the 2 sessions for each respective muscle and calculated a coefficient of variation (CV). The CV was calculated for each subject from the mean of each pre-conditioning measure between sessions and the corresponding standard deviation (CV = session standard deviation/session mean × 100).

To test whether the % change in MEP AUC was different between conditions, a separate ANOVA was performed for each muscle (APB, ED) with % change in MEP AUC as the dependent factor and CONDITION (EMG-triggered TMS, TMS-cued EMG) and TIME (post-0, post-15, post-30, post-45, post-60) as independent factors. To test whether the change in MEP AUC was different between muscles (APB or ED), a separate ANOVA was performed for each condition with % change in MEP AUC as the dependent factor and MUSCLE (APB, ED) and TIME (post-0, post-15, post-30, post-45, and post-60) as independent factors. To determine whether MEP AUC increased as a result of conditioning, a separate ANOVA was performed for each muscle (APB, ED) with absolute MEP AUC as the dependent factor and CONDITION (EMG-triggered TMS, TMS-cued EMG) and TIME (pre, post-0, post-15, post-30, post-45, and post-60) as independent factors. To determine whether changes in MEP AUC were different between the study muscles (APB, ED) and a control muscle (FCR) only for the TMS-cued EMG condition, a separate ANOVA was performed for each muscle (APB, ED) with % change in MEP AUC as the dependent factor and MUSCLE (study muscle, FCR) and TIME (post-0, post-15, post-30, post-45, post-60) as independent factors. Note that the data for the other control muscle, biceps, was uninterpretable in many subjects due to large stimulus artifacts in the EMG; biceps data was therefore omitted from the analysis.

For the RMT outcome measure ANOVA calculations were performed to assess for differences between conditions and decreases in RMT as a result of conditioning; a separate ANOVA was performed for each muscle (APB, ED) with RMT as the dependent factor and CONDITION (EMG-Triggered TMS, TMS-cued EMG) and TIME (pre, post-0, post-15, post-30, post-45, post-60) as independent factors. Further ANOVA calculations were performed on the CSP outcome measure to assess for differences between condition and increases in CSP post-conditioning for each muscle (APB, ED); for these calculations the dependent factor was CSP and independent factors were the same as for RMT ANOVA calculations.

The Holm-Bonferroni method was applied to all ANOVA statistics to correct for multiple comparisons and *P*-values were adjusted accordingly. The significance level was set to *P* < 0.05 unless otherwise stated in figure legends. Error bars represent 95% confidence intervals for standard error. All statistical analyses were performed with Matlab and Stata.

## Results

### Timing of TMS pulses in relation to EMG onset

**Figure 2:**
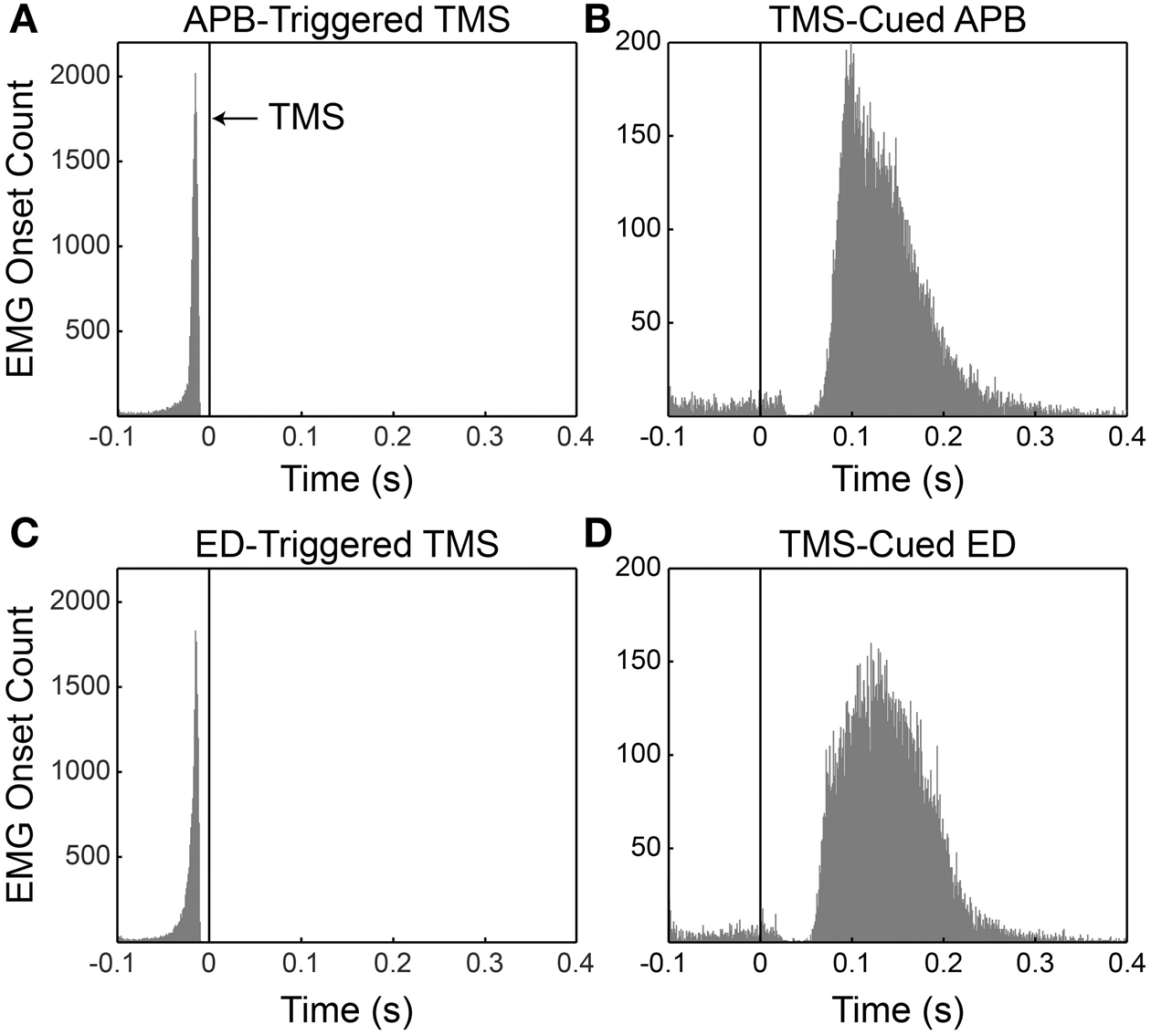
**Histograms of volitional EMG onset with respect to the timing of the TMS pulse for all subjects**. **(A)** APB-triggered TMS. **(B)** TMS-cued APB. **(C)** ED-triggered TMS. **(D)** TMS-cued ED. Black vertical lines at *t* = 0 denote occurrence of the TMS pulse. Note in panels B and D that instances in which EMG onset occurred between −100 and ~25 ms represent premature responses by the subject. The lack of premature responses between ~25 and ~50 ms in panels **B** and **D** likely reflects mild cortical inhibition (a very short cortical silent period) induced by the preceding TMS pulse.

The mean timing ± SD of EMG onset in relation to the TMS pulses (Figure 2) was −21.9 ms ± 16.7 ms for APB-Triggered TMS, −22.2 ms ± 16.1 ms for ED-Triggered TMS, 130 ms ± 65 ms for TMS-Cued APB, and 134 ms ± 61 ms for TMS-Cued ED.

TMS-evoked MEPs occurred routinely during EMG-triggered TMS and occasionally during TMS-cued EMG. These TMS-evoked MEPs were expected during EMG-triggered TMS sessions (see Figure 1) because motor activity reduces the motor threshold (Hess et al., 1987). We did not anticipate TMS-evoked MEPs during TMS-cued EMG sessions because stimulation intensity was below RMT and subjects relaxed the muscle of study before each ensuing stimulus. Nevertheless, TMS-evoked MEPs occurred for 20% of stimuli in the TMS-Cued APB session and 17.7% of stimuli in the TMS-Cued ED session. We suspect the rapid stimulation frequency (1 ± 0.1 Hz) led subjects to anticipate ensuing TMS pulses in preparation for muscle activation, thereby lowering RMT during TMS-cued EMG sessions.

Determining the timing of EMG onset in the TMS-cued APB and TMS-cued ED sessions was challenging when a TMS-evoked MEP occurred in close temporal relation to volitional EMG onset. A Matlab algorithm identified TMS-evoked MEPs and differentiated them from the onset of volitional EMG. In rare instances for the TMS-cued APB and TMS-cued ED sessions, EMG onset was buried in a TMS-evoked MEP; in these instances EMG onset was identified as the first time the EMG amplitude rose above 200 μV (TMS-cued APB) or 100 μV (TMS-cued ED). The total number of times EMG onset was buried in a TMS-evoked MEP ± SD was 5± 7.9 for TMS-cued APB and 6 ± 6.7 for TMS-cued ED. Subjects occasionally had very late responses (>400 ms post-TMS). The number of very late responses ± SD was 21.9 ± 29.5 for TMS-cued APB and 5.8 ± 5.2 for TMS-cued ED. The discrepancy in the average number of late responses can be attributed to one subject with many such responses during TMS-cued APB. The total number of stimuli ± SD delivered during conditioning was 1709 ± 54 for APB-triggered TMS and TMS-cued APB and 1720 ± 34 for ED-triggered TMS and TMS-cued ED.

### Within subject variability from session to session

The results for pre-conditioning AMT, RMT, MEP area, and CSP were similar between the 2 sessions for each respective muscle (*t*-test, *P* > 0.05). The variability from session to session was small for the pre-conditioning measures AMT, RMT, and CSP, and large for MEP area (see Table 2). There were no significant differences in variability based on muscle tested.

**Table 2:** **Coefficient of variation (CV) of pre-conditioning AMT, RMT, MEP AUC, and CSP duration**.

	**APB**	**ED**	**APB**	**ED**	**APB**	**ED**	**APB**	**ED**
	**AMT**	**AMT**	**RMT**	**RMT**	**MEP**	**MEP**	**CSP**	**CSP**
					**AUC**	**AUC**		
Between-session variability	3.7	3.8	3.1	3.1	27.4	25.3	13.0	9.0

*The between-session variability was calculated from 2 sessions for each muscle from each subject; the CV represents the median value from all 10 subjects*.

### MEP area under the curve pre- and post-conditioning

**Figure 3:**
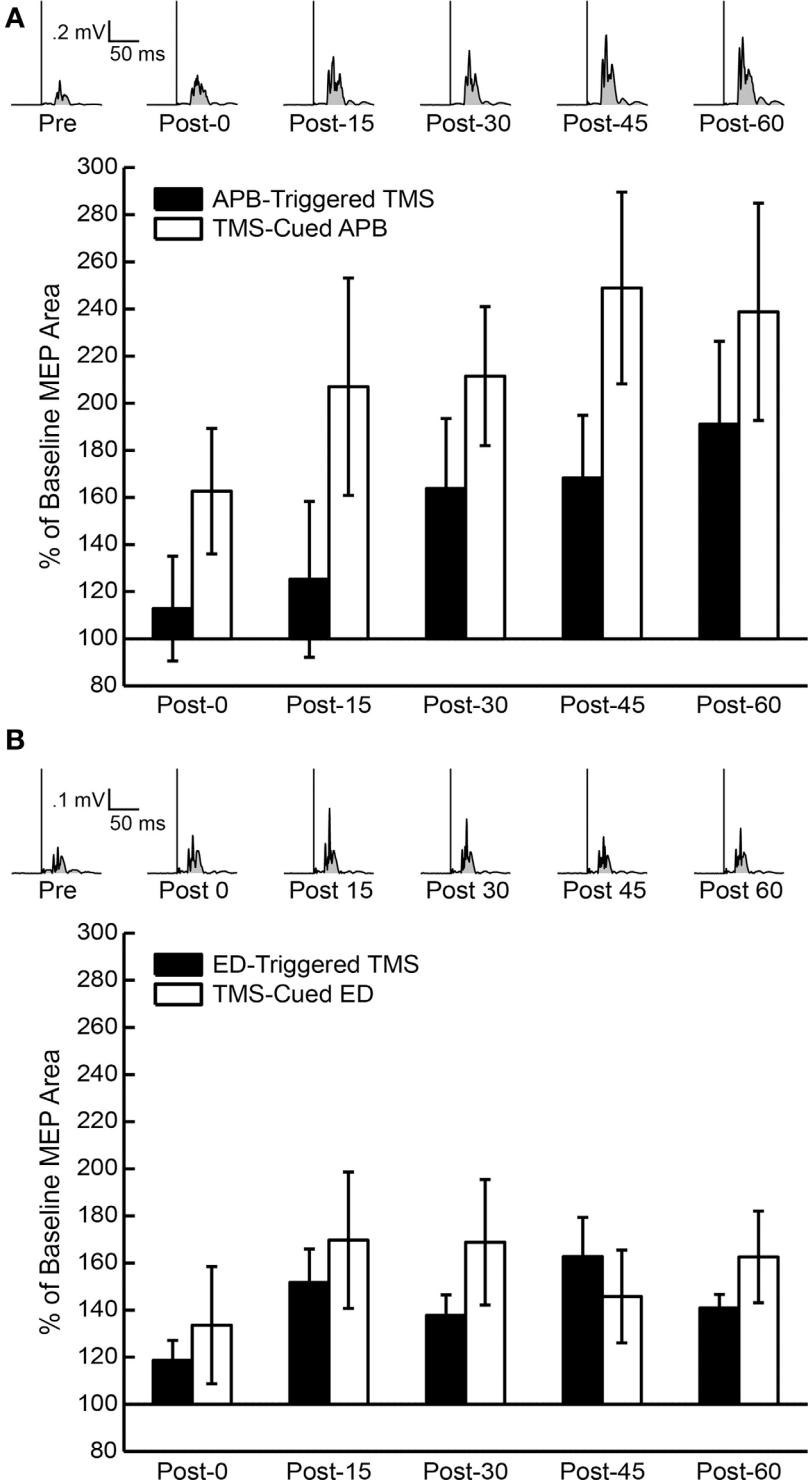
**Percent of baseline motor evoked potential (MEP) area under the curve (AUC) post-conditioning for all subjects**. **(A)** Average of 10 rectified MEPs at 120% resting motor threshold (RMT) for one representative subject's APB-Triggered TMS session (above) and the MEP AUC compared to pre-conditioning MEP AUC for all subjects following APB-Triggered TMS and TMS-Cued APB at post-0, post-15, post-30, post-45, and post-60 time periods (below). Shaded regions under rectified MEP's represent areas used for AUC measurements. **(B)** Average of 10 rectified MEPs at 120% RMT for one representative subject's TMS-cued ED session (above) and MEP AUC compared to pre-conditioning MEP AUC for all subjects following ED-Triggered TMS and TMS-cued ED at post-0, post-15, post-30, post-45, and post-60 time periods (below).

**Figure 4:**
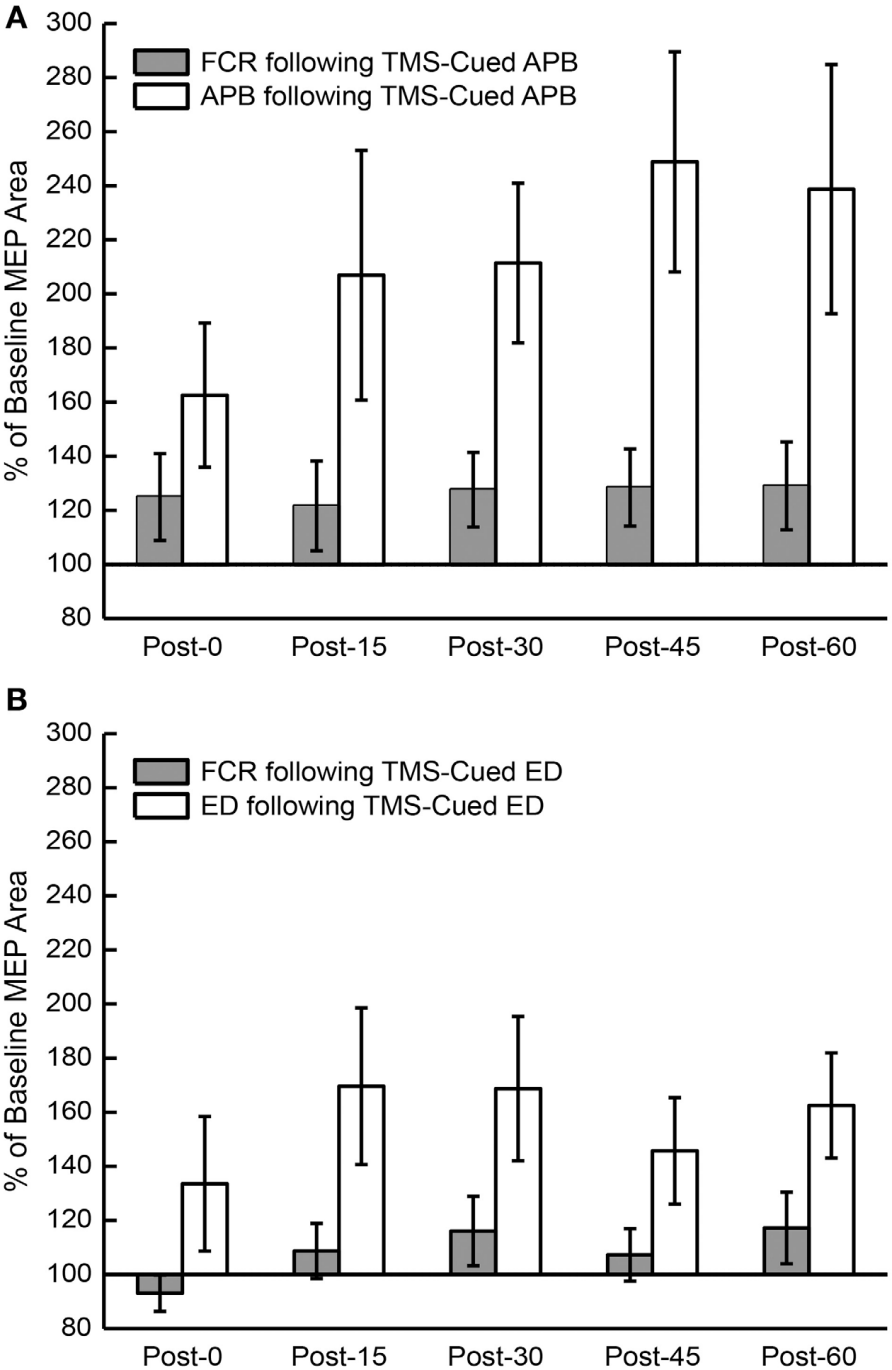
**Percent of baseline motor evoked potential (MEP) area under the curve (AUC) post-conditioning compared to a control muscle, flexor carpi radialis (FCR)**. **(A)** MEP AUC compared to pre-conditioning MEP AUC for FCR and APB following TMS-cued APB at post-0, post-15, post-30, post-45, and post-60 time periods. **(B)** MEP AUC compared to pre-conditioning MEP AUC for FCR and ED following TMS-cued ED at post-0, post-15, post-30, post-45, and post-60 time periods.

The mean % changes in MEP AUC for all sessions at each time period are displayed in Figure 3. ANOVA testing revealed no significant effect for CONDITION (EMG-Triggered TMS, TMS-cued EMG), TIME, or CONDITION X TIME on % change in MEP AUC for either the APB or ED muscles. Similarly, there was no significant effect for MUSCLE (APB, ED), TIME, or MUSCLE X TIME on % change in MEP AUC for either the EMG-triggered TMS or TMS-cued EMG condition. On the other hand, ANOVA testing that compared time points before and after conditioning showed a significant effect for TIME on the absolute MEP AUC, reflecting post-conditioning increases for both conditions and muscles tested [APB: *F*_(1, 5)_ = 5.93, *P* = 0.005; ED: *F*_(1, 5)_ = 5.86, *P* = 0.005]. The factors CONDITION and CONDITION X TIME were not significant. The % change in MEP AUC for the muscle of study (APB or ED) vs. a control muscle (FCR) solely for the TMS-cued sessions is displayed in Figure 4. ANOVA testing initially suggested increased % change in MEP AUC for each MUSCLE (APB or ED) in comparison to FCR, but this was not significant after correction for multiple comparisons; the factors TIME and MUSCLE X TIME were not significant. In summary, MEP AUC increased in response to all 4 conditioning sessions, with no significant difference between conditions or muscles tested.

### Active motor threshold pre-conditioning

Mean pre-conditioning AMT ± SE was 38.6 ± 1.6 for APB-triggered TMS, 37.8 ± 1.0 for TMS-cued APB, 37.7 ±1.5 for ED-triggered TMS, and 37.6 ± 1.6 for TMS-cued ED.

### Resting motor threshold pre- and post-conditioning

**Figure 5:**
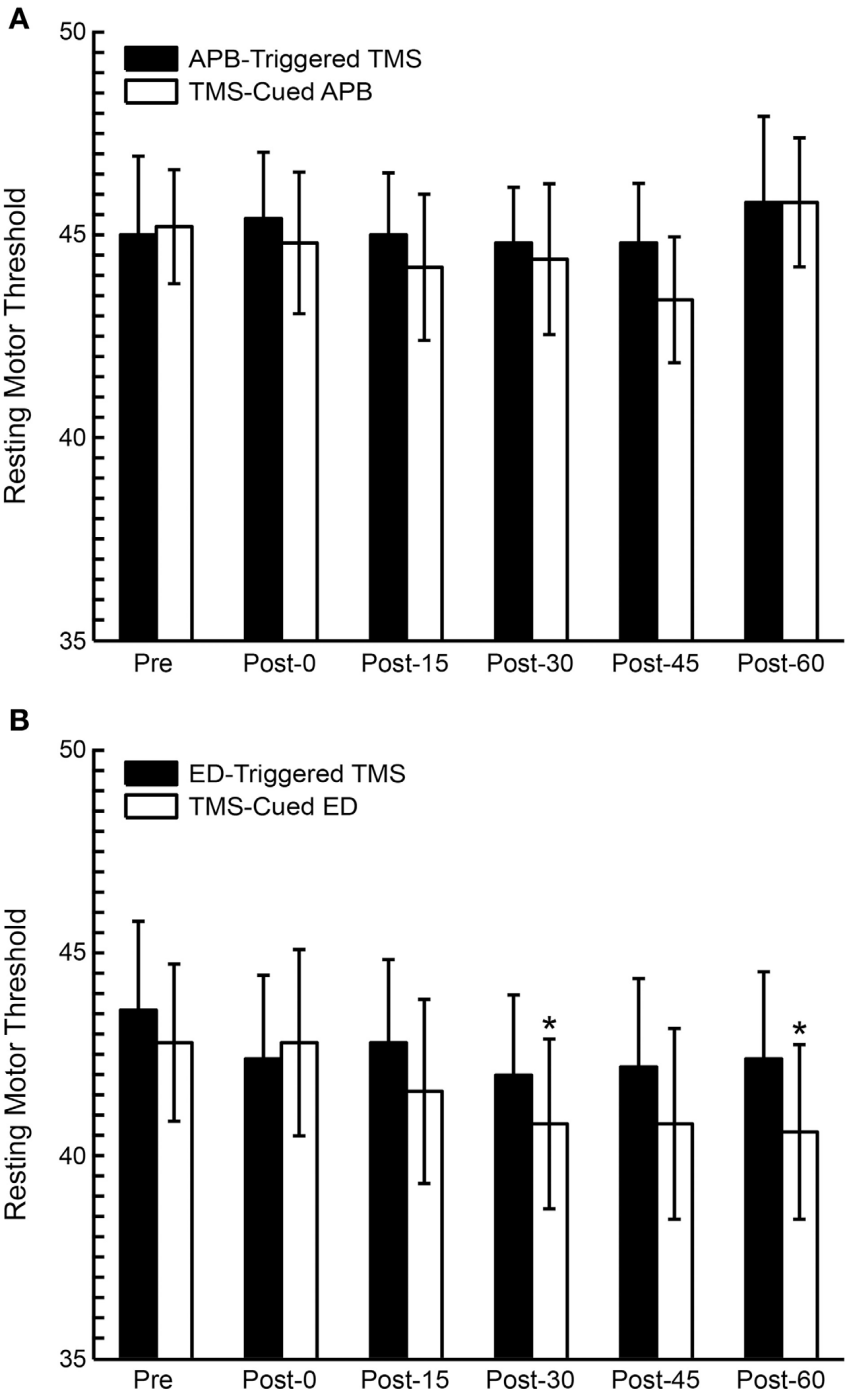
**Resting motor threshold (RMT) pre and post-conditioning for all subjects**. **(A)** RMT before and after APB-Triggered TMS and TMS-cued APB. **(B)** RMT before and after ED-Triggered EMS and TMS-cued ED. *T*-test, ^*^P < 0.05.

RMT was compared pre- and post-conditioning (Figure 5). ANOVA testing revealed no significant differences in RMT between CONDITIONS (EMG-Triggered TMS, TMS-cued EMG), but the factor TIME showed a general decrease in RMT post-conditioning that was significant for the ED muscle [*F*_(1, 5)_ = 4.66, *P* = 0.02]; there were no CONDITION X TIME interactions. Two specific time periods also showed significant decreases in RMT for the ED muscle in *post hoc* testing (see Figure 5), but only following TMS-cued muscle activity.

### Cortical silent period pre- and post-conditioning

**Figure 6:**
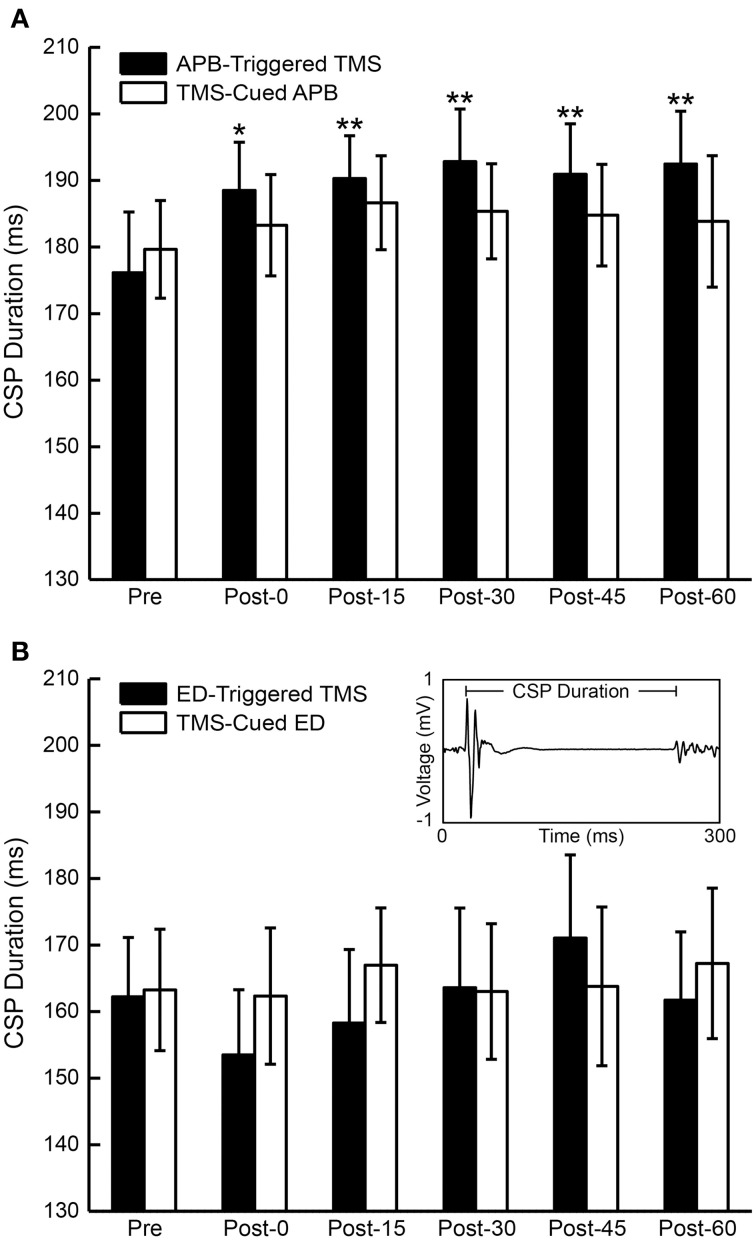
**Cortical silent period (CSP) duration for all subjects**. **(A)** The mean duration of the CSP before and after APB-Triggered TMS and TMS-Cued APB. **(B)** Mean duration of the CSP before and after ED-Triggered TMS and TMS-cued ED. *T*-test, ^*^P < 0.05, ^**^P < 0.01. Inset—motor evoked potential (MEP) and ensuing CSP from one representative subject showing CSP duration from MEP onset to the return of volitional EMG activity.

CSP duration was compared pre- and post-conditioning for each time period for all subjects (Figure 6). ANOVA testing revealed no significant differences in CSP between CONDITIONS (EMG-Triggered TMS, TMS-cued EMG). There was, however, a significant effect for TIME showing increase in CSP duration following APB-triggered TMS [*F*_(1, 5)_ = 5.25, *P* = 0.01] that was not observed for the other 3 conditions; there were no CONDITION X TIME interactions.

## Discussion

Previous studies of activity-dependent stimulation in animals and humans found that motor or neural activity occurring 0–50 ms before brain stimulation leads to LTP-like effects (Bütefisch et al., 2004; Jackson et al., 2006; Rebesco et al., 2010; Lucas and Fetz, 2013; Nishimura et al., 2013). In particular, intracranial microstimulation of a site representing a muscle systematically triggered from another muscle's activity modified the output effects evoked by subsequent cortical stimulation. We therefore hypothesized that repeated pairing of muscle activity occurring before TMS at the muscle's cortical site would cause greater increases in corticospinal excitability than muscle activity after TMS. Our results did not support this hypothesis, and suggest that brain stimulation delivered either shortly before or after muscle activity can increase MEP area, which may reflect increased corticospinal excitability.

Our unexpected finding of increased MEP area following both TMS-cued EMG and EMG-triggered TMS is difficult to explain in the context of prior studies. One possible explanation is that TMS-cued EMG can also induce LTP-like effects through Hebbian mechanisms. There is precedent for this in the work of Thabit and colleagues (Thabit et al., 2010), who demonstrated that pairing motor activity 50 ms after TMS increased corticospinal excitability. The rapid cue rate in our study probably allowed subjects to anticipate the timing of successive cues despite our efforts to mitigate premature responses by varying the cue frequency by ± 0.1 Hz. This anticipation likely shortened mean reaction times (Jakobs et al., 2009), allowing a significant proportion of motor activity to occur 50–100 ms post-TMS (see Figures 2B,D). In addition, studies show increased cortical activation up to 80 ms prior to EMG onset during reaction time tasks (Pascual-Leone et al., 1992; Chen et al., 1998). It follows that these early muscle responses and cortical activation well in advance of EMG onset could bring a significant fraction of trials in our TMS-cued EMG sessions within the temporal window for the potentiating effects described by Thabit et al. Like the findings by Thabit et al. the increased MEP area in our TMS-cued EMG sessions developed quickly (within 40 min) and continued for an extended period of time (1 h). Note that the stimulation intensities during conditioning for the present experiment (~85% RMT) were much lower than the intensity employed by Thabit and colleagues (120% RMT). This low stimulation intensity might explain why our study appeared underpowered to show differences in MEP area between the muscle of study and a control muscle and why CSP was not prolonged following all conditions in parallel with the MEP area changes. Higher stimulation intensities may be necessary to induce robust LTP-like effects.

Our results did not strictly meet our criteria for an LTP-like phenomenon given our inability to show specificity for the muscle of study and a seeming lack of dependence on the timing of stimulation. Nevertheless, the increase in MEP area seen with TMS-cued EMG could conceivably be explained by NMDA receptor-mediated STDP. *In vitro* studies suggest that synapses between two neurons are strengthened when the action potential (AP) of the presynaptic neuron is followed within 50 ms by an AP from a postsynaptic neuron (Markram et al., 1997; Bi and Poo, 1998; Feldman, 2000; Caporale and Dan, 2008). Both events are necessary to allow Ca^2+^ influx into the postsynaptic cell and create LTP—the presynaptic AP releases the excitatory neurotransmitter glutamate and the postsynaptic AP unblocks Mg^2+^ from the NMDA receptor (Nowak et al., 1984). We theorize that the potentiating effects observed with TMS-cued EMG fit this model as follows. Each TMS pulse would create multiple presynaptic APs in the stimulated cortical region, releasing glutamate into synaptic clefts. Volitional activity in M1 sufficient to activate muscles shortly thereafter would provide the back-propagating APs necessary to unblock NMDA receptors and allow Ca^2+^ influx.

One key difference in study design could help explain why our TMS-cued EMG results and the findings of Thabit et al. differ from the findings described by other investigators. We paired activity in a single muscle with stimulation to the cortical representation for the *same* muscle in M1 while other investigators paired muscle or neural activity with stimulation to the cortical representation for a *different* muscle in M1 (Bütefisch et al., 2004; Jackson et al., 2006; Lucas and Fetz, 2013). This difference could affect which neurons in M1 are primed for STDP at a given point in time, thereby affecting the timing rules for modifying horizontal connections between cortical neurons. The rules governing STDP are known to vary considerably between different modes of stimulation and for excitatory vs. inhibitory synapses (Sjöström et al., 2001; Caporale and Dan, 2008; Froemke et al., 2010).

The increase in MEP area seen following EMG-triggered TMS in the present study may also be explained by STDP. Theoretically, reversing the time course of events described above by using volitional activity to cause presynaptic APs and cortical stimulation within 50 ms to create back-propagating APs in postsynaptic cells might also lead to LTP. Indeed, this is the proposed mechanism of action for the potentiating effects seen in most prior activity-dependent stimulation protocols (Bütefisch et al., 2004; Jackson et al., 2006; Rebesco et al., 2010; Lucas and Fetz, 2013). Our time window of ~22 ms between EMG onset and TMS fell within the range for potentiating effects described by prior investigators. Animal studies of activity-dependent cortical stimulation suggest LTP-like effects with a short window (several ms) between EMG onset (Lucas and Fetz, 2013) or neural activity (Rebesco et al., 2010) and cortical stimulation; this time window may extend from 0–50 ms for cortically-triggered cortical stimulation (Jackson et al., 2006). The time windows for other human studies reporting neural plasticity with EMG-triggered TMS were 40 ms in one study (Bütefisch et al., 2011) and were unspecified in the other (Bütefisch et al., 2004). Given that neurons in non-human primate M1 continue to fire up to 250 ms with ballistic forelimb movement (Murphy et al., 1982) one might expect a potentially wide temporal window for neuroplasticity with EMG-triggered TMS; yet one human study suggests LTP-like effects occur only when the stimulus arrives during the motor execution phase of motor imagery (Mrachacz-Kersting et al., 2012). As with TMS-cued EMG, the increases in corticospinal excitability following EMG-triggered TMS developed quickly and lasted at least an hour.

One problem with citing STDP as a mechanism for the increased MEP area following both TMS-cued EMG and EMG-triggered TMS is the bidirectional nature of STDP depending on relative timing (Markram et al., 1997; Bi and Poo, 1998; Wolters et al., 2003; Thabit et al., 2010). Thus, when the timing of volitional activity or peripheral nerve stimulation in relation to brain stimulation is switched around the zero point, what once led to LTP-like effects then causes LTD-like effects. While we did not observe such temporal asymmetry, our experiment was also not designed to test a multitude of timing windows between TMS onset and the onset of volitional muscle activity. Future studies of activity-dependent stimulation in humans that vary the timing between TMS onset and muscle activity at 10–25 ms intervals may help clarify optimal time windows to induce LTP- and LTD-like effects.

Given our finding of increased MEP area following both TMS-cued EMG and EMG-triggered TMS, as well as the lack of temporal asymmetry, one may argue that our results stem from repeated muscle activation alone or 1 Hz TMS alone. We did not perform these control sessions; however, these explanations seem improbable for several reasons. Prior investigators testing the effects of repeated ballistic (Giesebrecht et al., 2012) and isometric (Samii et al., 1996) muscle contraction for 30 min and 10 min respectively in the absence of brain stimulation found that MEP amplitude increased 200–250% immediately post-exercise, but then returned to baseline levels within 10 min. Our findings of increased MEP AUC for up to an hour post-conditioning do not fit this pattern of short-lived changes. Subthreshold 1 Hz TMS alone appears even less likely to explain increases in corticospinal excitability. Prior studies demonstrated that prolonged subthreshold 1 Hz rTMS with the subject at rest either leads to no change (Siebner et al., 1999) or a 10 min period of decreased MEP amplitude (Touge et al., 2001; Romero et al., 2002) post-stimulation. As an aside, it is interesting to note that the trend toward an increase in MEP AUC from the post-0 to post-60 time periods in the present study might be explained by the brief inhibitory aftereffects of subthreshold 1 Hz TMS competing with the more durable potentiating effects of activity-dependent stimulation.

We employed a faster mean stimulation frequency during conditioning sessions (1 Hz) than prior investigators exploring the effects of activity-dependent stimulation in human subjects (Stefan et al., 2000; Bütefisch et al., 2004; Thabit et al., 2010) (0.1 Hz, 0.05 Hz, and 0.2 Hz respectively). We thought that more stimuli would provide greater cumulative plasticity, but sometimes subjects had difficulty keeping up with the pace, leading to errors in the form of early or missed muscle reactions to the visual cue. A mean stimulation frequency between 0.2–0.5 Hz may be better suited to maximize the opportunity for Hebbian-type plasticity and minimize errors. This may also improve translational efforts since patients with brain injury are likely to be slower with tasks requiring manual dexterity.

To our knowledge, this is the first human study to test the effects of activity-related cortical stimulation in two separate muscles. We found increased MEPs following TMS-cued EMG and EMG-triggered TMS for both muscles of study, though we were unable to show that these changes were significantly different than the control muscle FCR after correction for multiple comparisons. Nonetheless, these results are encouraging because they suggest it may be possible to target the potentiating effects of activity-dependent stimulation to muscles critical for recovery of function following brain injury. Prior studies targeting the extensor muscles of the distal upper limb with activity-dependent stimulation show mixed results. A protocol that included both TMS and peripheral neuromuscular stimulation in chronic stroke patients led to significant increases in corticospinal excitability, dexterity, and strength in the ED muscle post-conditioning (Koganemaru et al., 2010). On the other hand, a protocol with strong evidence of neural plasticity for the APB muscle with EMG-triggered TMS in healthy subjects (Bütefisch et al., 2004), had less robust effects when tried on the extensor carpi ulnaris (ECU) muscle of stroke patients in a separate experiment (Bütefisch et al., 2011). Whether this difference was due to corticospinal tract damage from stroke or to other neurophysiologic differences between the APB and ECU cortical organization remains unclear. One would expect issues of cortical organization to play little role considering that the corticomotoneuronal cells for the muscles of the distal upper limb are largely overlapping in the monkey (Rathelot and Strick, 2006; Smith and Fetz, 2009).

Although the increased CSP following APB-triggered TMS alone would seem to support our original hypothesis, this did not parallel changes to our other primary outcome measure of corticospinal excitability, MEP AUC. Investigators attribute changes in CSP duration to altered GABA_B_ergic inhibitory tone in the cortex (Werhahn et al., 1999; Pierantozzi et al., 2004). Most prior TMS studies causing LTP-like effects in M1 resulted in prolonged CSP (Stefan et al., 2000; Koganemaru et al., 2010; Thabit et al., 2010), while others found no change (Di Lazzaro et al., 2011) or shortened CSP (Khedr et al., 2007). Our results, suggesting increased CSP duration following EMG-triggered TMS alone are difficult to explain in the context of prior studies. As described earlier in the discussion, using suprathreshold stimulation intensities during conditioning could lead to CSP changes that better reflect MEP area results.

Future studies should address some of the limitations of this study. To determine whether changes in corticospinal excitability could be caused solely by repeated muscle activity it would be helpful to run a control group performing volitional muscle contractions alone during sham TMS. An additional control of 1 Hz rTMS with no volitional muscle activity might also be helpful. Although our ability to randomize study sessions was limited due to the necessity of an EMG-triggered session to obtain the stimulation times for the subsequent TMS-cued session, future studies could randomize study sessions by muscle to prevent potential unforeseen bias based on familiarity with the experimental protocol during later sessions. We tested changes in MEP AUC using a stimulation intensity of 120% RMT; while this resulted in high pre-conditioning variability from session to session, this was no different than the variability in MEP AUC reported by other investigators at similar intensity (Van Der Kamp et al., 1996). Note that between-session variability in MEP area decreases with increasing stimulator intensity (Van Der Kamp et al., 1996) and generating full stimulus-response curves (Malcolm et al., 2006) before and after conditioning might lead to different results at different stimulation intensities. Possible functional outcome measures, such as voluntary muscle force, should also be assessed. Potential spinal involvement could be tested by measuring F-waves or the H-reflex. We assume that any changes in corticospinal excitability took place in the cortex, but cannot exclude the possibility that some changes occurred in the spinal cord. To provide more information on activation of inhibitory circuits in the motor cortex future studies could measure short-interval cortical inhibition, short-latency afferent inhibition, or long-latency afferent inhibition. The rapid frequency of the reaction time task during conditioning led to some behavioral errors in the form of early or late muscle responses, which could be addressed by longer intervals between trials.

Despite these limitations, the results from this study will inform future activity-dependent stimulation protocols. By delivering TMS from a recording in the TMS-cued APB and TMS-cued ED sessions, we ensured that subjects received exactly the same number of TMS pulses with identical timing in the two conditioning sessions for each respective muscle of study. We showed that pairing muscle activity either before or after brain stimulation can increase MEP area; however, the optimal timing between brain stimulation and muscle activity to maximize gains remains to be elucidated. Future studies with a larger sample size, testing multiple intervals between EMG onset and brain stimulation may ultimately help to resolve this timing mystery. Activity-dependent stimulation continues to hold great promise for clinical rehabilitation. To our knowledge, this is the first human study attempting to increase corticospinal excitability in the motor representation for two separate muscles. Our results showing increases in MEP area for both muscle representations suggest activity-dependent stimulation protocols may be well suited to target specific motor cortical areas for Hebbian-type plasticity following brain injury.

### Conflict of interest statement

The authors declare that the research was conducted in the absence of any commercial or financial relationships that could be construed as a potential conflict of interest.
